# Cutaneous Metastasis of Triple Negative Breast Cancer: A Case Report and Review of Literature

**DOI:** 10.7759/cureus.79926

**Published:** 2025-03-02

**Authors:** Stefanos Flindris, Chrysoula Margioula-Siarkou, Georgia Margioula-Siarkou, Chrysoula Gouta, Effrosyni Styliara, Iordanis Navrozoglou, Stamatios Petousis, Konstantinos Dinas

**Affiliations:** 1 2nd Department of Obstetrics and Gynecology, Gynecologic Oncology Unit, Ippokrateio General Hospital of Thessaloniki, Aristotle University of Thessaloniki, Thessaloniki, GRC; 2 Department of Pathology, Hippokrateio General Hospital, Thessaloniki, GRC; 3 Department of Radiology, University Hospital of Ioannina, Ioannina, GRC; 4 Department of Obstetrics and Gynecology, University of Ioannina, Ioannina, GRC

**Keywords:** breast cancer, cutaneous, metastasis, treatment, triple negative

## Abstract

Cutaneous metastases (CMs) from breast cancer are rare but represent a significant manifestation of advanced disease, occurring in a small group of patients. They often present metachronously and may resemble dermatologic conditions, complicating diagnosis.

We report a 95-year-old woman with a history of ductal triple negative breast cancer (TNBC), initially treated with mastectomy and radiotherapy. Eight years post-mastectomy, she developed an erythematous and indurated skin lesion in the left axilla and one in the rachis. Histopathology confirmed metastatic carcinoma of the breast and CT scan revealed also pulmonary metastasis. Palliative treatment with topical imiquimod and supportive care was decided as the best treatment option due to the patient’s very advanced age and comorbidities. The patient remains stable six months post-diagnosis.

CMs indicate metastatic disease and require prompt recognition to avoid misdiagnosis. Dermatoscopy and biopsy with histological confirmation are the cornerstone tools for the diagnostic assessment. Surgical resections may be ideal for localized disease, but topical and/or systemic therapy is an effective alternative. Palliative care in combination with topical agents’ administration offers symptom relief and maintains quality of life in the over elderly patients.

Early recognition of CMs is crucial for accurate diagnosis and optimal management. A multidisciplinary approach ensures timely intervention, while treatment should be tailored to balance efficacy and quality of life, precisely in elderly patients.

## Introduction

Breast cancer represents the most common malignancy in women [1]. A rare form of breast cancer metastasis or recurrent disease is cutaneous metastasis (CM), which represents 20% of all skin metastases and is presented in approximately 2.4% of metastatic breast cancer patients [2]. Frequently, CM occurs after the diagnosis of primary malignancy and after a long follow-up interval within 10 years from the initial management [3,4]. An exception is the inflammatory breast cancer which represents a unique entity of the disease [5]. The manifestation of skin metastases signifies systemic disease and a poor prognosis [5,6]. The wide range of symptoms may include single or multiple erythema, diffuse sclerosis, eczema, rashes, painless or painful, firm and indurated lesions of the skin [7,8]. Lesions may also appear as seed-like papules and even larger lesions [9]. Commonly affected areas are the breast as a local recurrence of the disease or distant skin areas in the chest wall, the back, the upper extremities, the neck, the scalp, and the abdomen [7,9,10].

The recognition and evaluation of cutaneous metastatic disease after breast cancer surgery can be perplexing due to its clinical resemblance to other dermatological maladies such as cellulitis and lymphedema [11]. Usually, dermatologists may be the first to recognize the lesions by clinical evaluation and dermatoscopy, which is used to differentiate between skin metastases and other benign or malignant dermatologic conditions [9,12,13]. However, the dermatoscopic patterns of skin metastases have not been established comprehensively yet [9,12]. For definite diagnosis skin biopsy and pathology evaluation is essential to set the diagnosis [14]. We report a case of an elderly woman with CM from triple negative breast cancer (TNBC) eight years after the initial diagnosis and treatment.

## Case presentation

The case refers to a 95-year-old patient, G4P3A1 who had been in menopause for 50 years. She denied alcohol abuse, smoking, and she reported coronary disease treated with stents at the age of 88, osteoporosis, and history of deep venous thrombosis in the lower extremity. Eight years ago, at the age of 87, the patient was diagnosed with ductal breast carcinoma at the right breast 5.5x3cm in size. She underwent a modified radical mastectomy with axillary lymphadenectomy revealing a basal like ductal breast cancer, grade 3, pT3N2aM0 with 5+/13 positive axillary lymph nodes. The immunohistochemistry stated ER, PR and HER2 negativity and Ki-67%:15%-30%. The multidisciplinary oncological meeting decided adjuvant radiotherapy but not complementary chemotherapy because of her extreme age and comorbidities. The radiotherapy dose was 42.56 Gy divided in 16 sessions in the right hemithorax, and 44 Gy divided in 20 sessions in the unilateral supraclavicular region. An additional dose of 7.5-12.5 Gy in three to five sessions targeting the skin of the operated region based on START trials.

**Figure 1:**
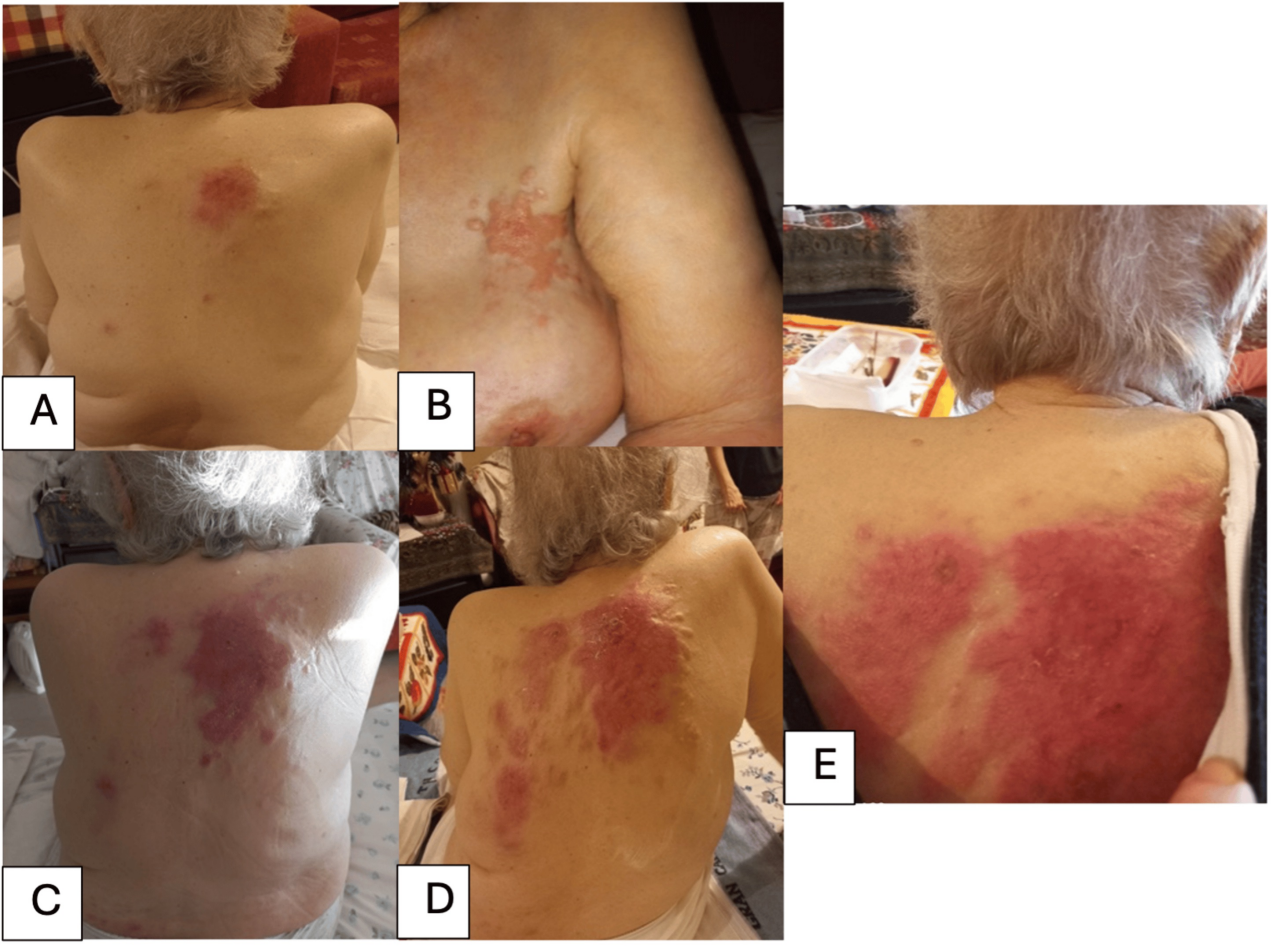
(A, B) Initial presentation of cutaneous metastases from triple-negative breast cancer (TNBC) demonstrating early lesions in the left axilla-breast region and a localized nodule on the back. (C) Rapid progression at 15-day follow-up, with elongation of the dorsal lesion. (D) By day 21, the lesion on the back becomes diffuse highlighting TNBC's aggressive behavior. (E) One-month post-diagnosis, extensive metastasis involves nearly the entire rachis, underscoring the critical need for early detection and intervention in TNBC cases.

After a disease-free period of eight years, she referred to a dermatologist to examine a rapidly growing erythematous, indurated and painless skin lesion that started from the other counterpart (left) breast-axilla and the back (Figures 1A, 1B). The physical examination revealed a diffuse well-demarcated erythematous macules and erysipelas-like skin spreading gradually in one month interval to the whole back of the patient (Figures 1C-1E.) Dermoscopic findings revealed pink to red ground, linear irregular and polymorphic vessels, shiny white homogenous areas and irregular fissure-like depressions.

To differentiate CMs from other skin conditions, clinicians should assess key features such as lesion morphology, distribution, and progression [14]. CMs often present as rapidly growing, firm, painless dermal or subcutaneous nodules, sometimes with telangiectasias or an indurated plaque-like appearance [14]. Unlike primary skin tumors or inflammatory dermatoses, they usually have intact overlying epidermis without ulceration unless advanced. Certain cancers exhibit characteristic patterns, breast cancer may present with carcinoma erysipeloides (CE) (resembling cellulitis) or “en cuirasse” (armor-like thickening), while melanoma often appears as multiple nodules due to angiotropism [13,14]. Metastatic lesions may mimic benign conditions like cysts, lipomas, or dermatofibromas but grow more aggressively and are diffused [10,15]. Clinical suspicion should be heightened in patients with a known malignancy or unexplained skin lesions. A biopsy (punch or excisional) is crucial for confirmation, along with imaging and histopathology to determine the primary source and guide treatment [16].

**Figure 2:**
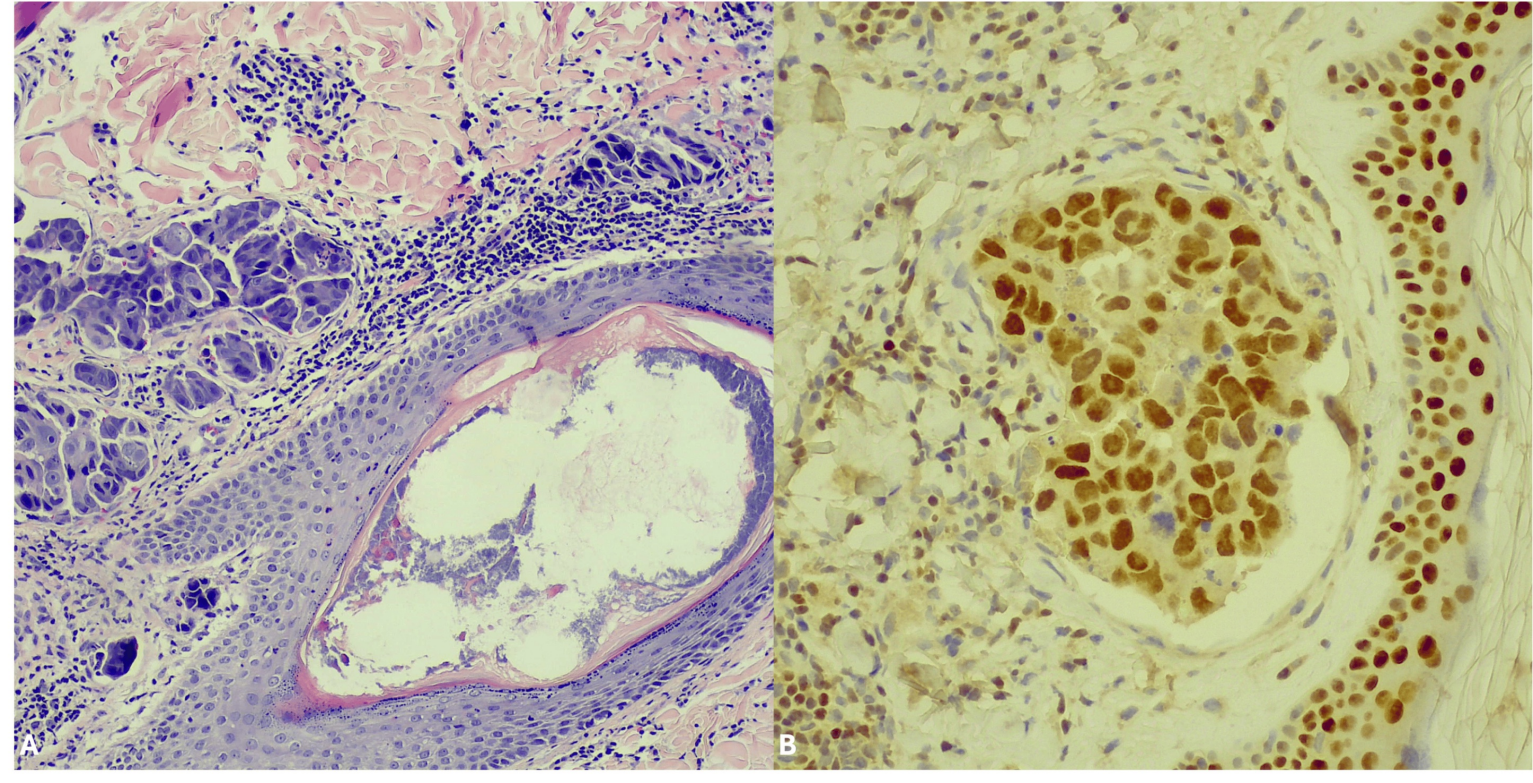
(A) Multiple metastatic foci in the lamina propria, adjacent to the skin adnexa (10x magnification). (B) GATA-3 nuclear positivity in neoplastic cells (20x magnification).

The patient was referred to our department for further investigation and management. Dermal biopsies were taken from the skin lesions and sent to the Pathology Department of Ippokratio Hospital for further evaluation. Microscopic examination revealed extended infiltration of papillary and upper reticular lamina propria with neoplastic epithelioid cells arranged in nest, sheets or rarely as individual cells intermingled between collagen fibers (Figure 2A). Immunohistochemical examination showed positivity of the neoplastic cells for GATA-3 (Figure 2B). Based on the morphological and immunohistochemical findings and the clinical history of the patient, the diagnosis of metastatic invasive breast carcinoma of no special type (NST) to the skin was established. A computerized tomography (CT) scan of the thorax diagnosed interstitial infiltration and scattered nodules as well as enlarged mediastinal and supraclavicular lymph nodes. In contrast, the abdominal ultrasound and cranial CT showed no abnormalities. The patient’s condition and age contraindicated the systematic chemotherapy, and only palliative treatment was offered consisted of Aldara 5% cream once daily, optimized pain control management and dietitian support commenced high calorie shakes and emulsions. The patient is still alive, in follow-up without significant progress on the disease six months after the diagnosis of her recurrence.

## Discussion

CMs may be related with all cancer types with incidence of 0.7% to 10.4% of all cases and may represent the first sign of cancer relapse or clinically silent cancer [15]. Skin metastases may be observed synchronously at the time of cancer diagnosis or commonly metachronously [16]. They are mostly observed in elderly women as in our case and indicate noteworthy impact on significantly decreasing survival rates [16,17]. Estimated rate of skin metastasis from breast cancer is 2.42% [18]. Adenocarcinomas represent 60% of metastatic cancers in skin [3,19,20]. Apart from breast cancer, other malignancies that metastasize in the skin are lung cancer, gastrointestinal tract cancers, and hematopoietic malignancies [13,16].

Dermatoscopy may be used in order to differentiate breast cancer metastasis from other types of cancer skin metastases or benign dermatological conditions. However, definitive diagnosis may be given by tissue biopsy and histopathology accompanied by immunohistochemistry report [9,14,20,21]. Breast cancer immunohistochemistry reveals the CK7+/CK20- cytokeratin pattern [14]. Kitamura et al. and Rosendahl et al. describe certain dermatoscopic findings which would be pathognomonic to recognize the metastatic skin lesions such as polymorphous and atypical vessels as well as tendency for forming linear fissure-like structures, with small lateral depressions [12,22]. Furthermore, Kelati et al. signify the prognostic value of these polymorphic vessels [9].

Most CMs appear as solitary or multiple, ranging from nodules to widespread compact skin lesions, flesh-coloured nodules in the skin or subcutaneous layers, varying in size, and are usually painless [23]. CMs of a breast carcinoma may be classified as erysipeloid carcinoma, teleangiectatic carcinoma, carcinoma en cuirasse, neoplastic alopecia, zostemifrom metastasis and others [17,24,25]. Based on retrospective reports, the most common clinicopathological presentations of breast cancer skin metastasis are skin papules and/or modules found in 80% of patients, telangiectatic carcinoma in 11%, erysipeloid in 3%, en cuirasse in 3%, alopecia neoplastica in 2%, and zosteriform type in 0.8% [25-27]. Al Ameer et al. described a case of CE associated with underlying breast carcinoma, which was initially misdiagnosed and treated as eczematous dermatitis for two months [28]. CE is characterized by a rapid onset and aggressive progression, necessitating prompt diagnosis and early intervention to enhance survival outcomes [21,28]. The current case was classified as erysipeloid presentation, which is based on literature, one of the less frequent patterns.

Surgical excision offers a viable treatment approach for small and early detected cutaneous metastasis in patients with good performance status and not over elderly, with wide resection margins if there are attainable, because otherwise positive margins correlate with treatment failure [29]. However, rapid recurrence raises concerns potentially associated with the activation of dormant cancer cells during local therapy [30]. Standardized excision guidelines have not yet formed and patients with prior mastectomy or reconstructive surgery pose unique challenges, or with distant CM may face extensive dermal defects and the more complex closure will be necessary [31]. Advancements in plastic surgery techniques, such as thoracoabdominal flap, pedicled latissimus dorsi musculocutaneous or pedicled rotation flap have demonstrated utility in facilitating broader resections with reduced morbidity [32-34]. In addition, some authors support the adjuvant radiotherapy after the combination of metastasectomy and reconstructive technique for wound closure [3,32,35]. 

Surgical intervention is less advantageous in patients with concurrent visceral and CM because they halt the systemic therapies for a long interval preoperatively and risk disease progression [31,36,37]. For patients with confined localized CM and concurrent pulmonary metastasis, a combined metastasectomy procedure may be beneficial, when cytoreduction is maximized [29,31]. Especially, operable CM resection indicates favorable long term survival in retrospective data [38,39]. Accessible lesions may be managed with elliptical excision and primary closure, while larger defects prior to mastectomy may require flaps and grafts [7]. However, more extensive skin involvement typically precludes surgery. Carefully selected patients for operation are associated with low morbidity and may enhance the overall survival of those patients [4,31].

CMs of breast cancer indicate advanced metastatic disease and potential resistance to systemic treatments, especially when appearing in distant regions and extensively [24,27,40,41]. This often necessitates specialized skin and wound management from a plastic surgeon and a dermatologist [1,24]. Exacerbating skin disease can decrease dramatically the quality of life and impose an extremely negative psychological impact on the patient, leading to emotional depression and isolation [1]. Hence, the early recognition of dubious lesions at an early stage is crucial for the treatment plan, as surgical excision of limited lesion may be feasible only in case of early detection but may be unresectable in advanced stages [9,16,26]. Other treatments include local ointments as imiquimod, cryotherapy, radiotherapy, photosensitizers, oxygen flow assisted topical administration of methotrexate (OFAMTX), aromatase inhibitors, immunomodulators, systematic chemotherapy and targeted therapies as trastuzumab and pertuzumab for HER2 positive tumors, and small-molecule tyrosine kinase inhibitors (TKIs), such as lapatinib, tukatinib, and neratinib or combination of them [17,24,41-45].

Regarding their prognosis, CMs from breast cancer do not necessarily have as poor a prognosis as CMs from other internal malignancies [13]. According to Mayer et al., CMs from other internal malignancies carry a 4.3-fold increased relative risk of mortality compared with CMs from breast cancer [46]. Palliative treatment may be provided to patients with unresectable tumors [16]. This involves maintaining the lesion dry and clean and debriding it if bleeding occurs. To prevent infection spread, a hydrocolloid dressing is used [24]. Recent advances in TNBC reported that PIK3CA mutations have a high prevalence in this subtype of BC [47]. Alpelisib is an FDA approved selective PI3K inhibitor which has demonstrated efficacy in treated PIK3CA-mutated HR+/HER- disease BC according to SOLAR-1’ phase III randomized trial results and might be another treatment option for advanced stages [43,48]. Furthermore, an antibody drug conjugates (ADC), fam-trastuzumab deruxtecan (T-DXd) has good effects in HER2 low patients with advanced breast cancer according to phase III Destiny-Breast04 Trial [43,49,50].

Apart from CMs, our patient was also diagnosed with lung metastasis. Lung is often the first site of tumor relapse after surgery. The most frequently seen pattern is that of pleural metastasis, followed by hilar and/or mediastinal lesions and pulmonary nodules, as observed in this case [17,23]. It is estimated that 60%-74% of patients who die of breast cancer also had pulmonary metastases, with the lung as the sole site of metastases in 21% of cases [15]. Furthermore, as also observed in our case, even if the patient is set in palliative care, which might be reasonable, especially for the over elderly patients, palliative care may permit a rather stable disease with acceptable prolongation of life with good quality-of-life health [41].

Diagnosing CM lesions requires a high level of clinical suspicion due to their often subtle and confusing presentation. Although cutaneous metastases from internal malignancies are relatively rare in clinical practice, it is crucial to consider this possibility in any newly discovered lesions, regardless of their seemingly benign appearance. Early recognition, particularly in breast cancer cases, allows for a swift and accurate diagnosis and timely treatment [27,51].

## Conclusions

In conclusion, our case emphasizes that identifying CM is of great importance for the diagnosis, staging and prognosis. Dermatology assessment may play a fundamental role for early diagnosis and should always be attentive to the challenges of early detection, diagnosis and treatment. Patients should be educated to recognize early any suspicious lesions and promptly consult their breast surgeon to develop a therapeutic plan. Finally, it is important also to emphasize that prognosis of CM may not always be detrimental, especially in over elderly patients, and optimal management should be tailored to the patient balancing not only therapeutic effect but also quality of life.

## References

[1] Damaskos C, Dimitroulis D, Pergialiotis V (2016). An unexpected metastasis of breast cancer mimicking wheal rush. G Chir.

[2] Kalmykow B, Walker S (2011). Cutaneous metastases in breast cancer. Clin J Oncol Nurs.

[3] Weinmann S, Leo MC, Francisco M (2020). Validation of a ductal carcinoma in situ biomarker profile for risk of recurrence after breast-conserving surgery with and without radiotherapy. Clin Cancer Res.

[4] Hosseinpour R, Yavari Barhaghtalab MJ (2021). Cutaneous metastasis vs. isolated skin recurrence of invasive breast carcinoma after modified radical mastectomy. Case Rep Dermatol Med.

[5] Qin R, Wang X, Fan T, Wu T, Lu C, Shao X, Yin L (2024). Bilateral inflammatory recurrence of HER-2 positive breast cancer: a unique case report and literature review. Front Oncol.

[6] Rehman S, Naveed MA (2020). Skin metastasis in breast cancer patients; a case series. J Cancer Allied Spec.

[7] Hu SC, Chen GS, Lu YW, Wu CS, Lan CC (2008). Cutaneous metastases from different internal malignancies: a clinical and prognostic appraisal. J Eur Acad Dermatol Venereol.

[8] Handa U, Kundu R, Dimri K (2017). Cutaneous metastasis: a study of 138 cases diagnosed by fine-needle aspiration cytology. Acta Cytol.

[9] Kelati A, Gallouj S (2018). Dermoscopy of skin metastases from breast cancer: two case reports. J Med Case Rep.

[10] Lee HJ, Lim HS, Ki SY, Lee JE, Lee JS, Park MH (2020). Cutaneous scalp metastases of malignant phyllodes tumor of the breast. J Breast Cancer.

[11] Schwartz RA, Wiederkehr M, Lambert WC (2004). Secondary mucinous carcinoma of the skin: metastatic breast cancer. Dermatol Surg.

[12] Kitamura S, Hata H, Homma E, Aoyagi S, Shimizu H (2015). Pigmented skin metastasis of breast cancer showing dermoscopic features of malignant melanoma. J Eur Acad Dermatol Venereol.

[13] Alcaraz I, Cerroni L, Rütten A, Kutzner H, Requena L (2012). Cutaneous metastases from internal malignancies: a clinicopathologic and immunohistochemical review. Am J Dermatopathol.

[14] Wong CY, Helm MA, Kalb RE, Helm TN, Zeitouni NC (2013). The presentation, pathology, and current management strategies of cutaneous metastasis. N Am J Med Sci.

[15] Bittencourt Mde J, Carvalho AH, Nascimento BA, Freitas LK, Parijós AM (2015). Cutaneous metastasis of a breast cancer diagnosed 13 years before. An Bras Dermatol.

[16] Betlloch-Mas I, Soriano-García T, Boira I, Palazón JC, Juan-Carpena G, Sancho-Chust JN, Chiner E (2021). Cutaneous metastases of solid tumors: demographic, clinical, and survival characteristics. Cureus.

[17] Cho J, Park Y, Lee JC, Jung WJ, Lee S (2014). Case series of different onset of skin metastasis according to the breast cancer subtypes. Cancer Res Treat.

[18] Malvezzi M, Bertuccio P, Levi F, La Vecchia C, Negri E (2014). European cancer mortality predictions for the year 2014. Ann Oncol.

[19] da Costa RE, Dos Reis CA, Moura RD, Araújo AL, de Oliveira FT, Vieira SC (2021). Cutaneous metastasis of occult breast cancer: a case report. Pan Afr Med J.

[20] Tiodorovic D, Stojkovic-Filipovic J, Marghoob A (2024). Dermatoscopic patterns of cutaneous metastases: a multicentre cross-sectional study of the International Dermoscopy Society. J Eur Acad Dermatol Venereol.

[21] Hussein MR (2010). Skin metastasis: a pathologist's perspective. J Cutan Pathol.

[22] Rosendahl C, Cameron A, Tschandl P, Bulinska A, Zalaudek I, Kittler H (2014). Prediction without pigment: a decision algorithm for non-pigmented skin malignancy. Dermatol Pract Concept.

[23] Alani A, Roberts G, Kerr O (2017). Carcinoma en cuirasse. BMJ Case Rep.

[24] Shrivastava N, Balasubramanian A (2023). Cutaneous metastasis in breast cancer: a case series. Cureus.

[25] Cebeci D, Yaşar Ş, Güneş P, Aytekin S (2020). Telangiectatic carcinoma - like lymphangioma circumscriptum. A rare form of cutaneous metastasis of breast carcinoma: case report. Med Arch.

[26] El Khoury J, Khalifeh I, Kibbi AG, Abbas O (2014). Cutaneous metastasis: clinicopathological study of 72 patients from a tertiary care center in Lebanon. Int J Dermatol.

[27] Di Micco R, Santurro L, Gasparri ML (2019). Rare sites of breast cancer metastasis: a review. Transl Cancer Res.

[28] Al Ameer A, Imran M, Kaliyadan F, Chopra R (2015). Carcinoma erysipeloides as a presenting feature of breast carcinoma: a case report and brief review of literature. Indian Dermatol Online J.

[29] Otsuka I (2019). Cutaneous metastasis after surgery, injury, lymphadenopathy, and peritonitis: possible mechanisms. Int J Mol Sci.

[30] Alizadeh N, Mirpour H, Azimi SZ (2018). Scalp metastasis from occult primary breast carcinoma: a case report and review of the literature. Int J Womens Dermatol.

[31] Huang S, Parekh V, Waisman J (2022). Cutaneous metastasectomy: is there a role in breast cancer? A systematic review and overview of current treatment modalities. J Surg Oncol.

[32] Xu H, Liang Y, Tang W, Du X (2024). Combination of radiotherapy and flap reconstruction for cancer treatments (review). Mol Clin Oncol.

[33] Shinden Y, Nagata A, Nomoto Y (2020). Surgical resection with pedicled rotation flap for post-mastectomy locoregional breast cancer recurrence. Anticancer Res.

[34] Motono N, Shimada K, Kamata T, Uramoto H (2019). Sternal resection and reconstruction for metastasis due to breast cancer: the Marlex sandwich technique and implantation of a pedicled latissimus dorsi musculocutaneous flap. J Cardiothorac Surg.

[35] Haviland JS, Owen JR, Dewar JA (2013). The UK Standardisation of Breast Radiotherapy (START) trials of radiotherapy hypofractionation for treatment of early breast cancer: 10-year follow-up results of two randomised controlled trials. Lancet Oncol.

[36] Chraiet N, Zenzri Y, Bouaziz H (2020). Generalized cutaneous metastases of breast cancer: an uncommon presentation. Clin Case Rep.

[37] Li ZH, Wang F, Zhang P, Xue P, Zhu SJ (2022). Diagnosis and guidance of treatment of breast cancer cutaneous metastases by multiple needle biopsy: a case report. World J Clin Cases.

[38] Ueno T (2022). Surgical management of metastatic breast cancer: a mini review. Front Oncol.

[39] Pastorino U, Buyse M, Friedel SG (2018). General thoracic surgery long-term results of lung metastasectomy: prognostic analyses based on 5206 cases. https://www.semanticscholar.org/paper/GENERAL-THORACIC-SURGERY-LONG-TERM-RESULTS-OF-LUNG-Pastorino-Buyse/e168efcd14d7c99939b37fe79ae989a658b37e34.

[40] Starace M, Carpanese MA, Pampaloni F (2022). Management of malignant cutaneous wounds in oncologic patients. Support Care Cancer.

[41] Krishnasamy SR, Almazan TH, Suero-Abreu GA, Jung JY (2018). Successful treatment of cutaneous metastatic breast cancer with topical treatments that potentially synergize with systemic therapy: a case series. JAAD Case Rep.

[42] Salazar LG, Lu H, Reichow JL (2017). Topical imiquimod plus nab-paclitaxel for breast cancer cutaneous metastases: a phase 2 clinical trial. JAMA Oncol.

[43] Gámez-Chiachio M, Sarrió D, Moreno-Bueno G (2022). Novel therapies and strategies to overcome resistance to anti-HER2-targeted drugs. Cancers (Basel).

[44] Henriques L, Palumbo M, Guay MP, Bahoric B, Basik M, Kavan P, Batist G (2014). Imiquimod in the treatment of breast cancer skin metastasis. J Clin Oncol.

[45] Elodie GJ, Pascale G, Marie-Annick Q (2020). Cutaneous Breast Cancer Metastases Successfully Treated Using an Oxygen Flow Assisted Topical Administration of Methotrexate (OFAMTX). Dermatol Ther (Heidelb).

[46] Mayer JE, Maurer MA, Nguyen HT (2018). Diffuse cutaneous breast cancer metastases resembling subcutaneous nodules with no surface changes. Cutis.

[47] Rugo HS, André F, Yamashita T (2020). Time course and management of key adverse events during the randomized phase III SOLAR-1 study of PI3K inhibitor alpelisib plus fulvestrant in patients with HR-positive advanced breast cancer. Ann Oncol.

[48] Martínez-Sáez O, Chic N, Pascual T (2020). Frequency and spectrum of PIK3CA somatic mutations in breast cancer. Breast Cancer Res.

[49] Schreiber AR, O'Bryant CL, Kabos P, Diamond JR (2023). The emergence of targeted therapy for HER2-low triple-negative breast cancer: a review of fam-trastuzumab deruxtecan. Expert Rev Anticancer Ther.

[50] Modi S, Saura C, Yamashita T (2020). Trastuzumab deruxtecan in previously treated HER2-positive breast cancer. N Engl J Med.

[51] Putra HP, Djawad K, Nurdin AR (2020). Cutaneous lesions as the first manifestation of breast cancer: a rare case. Pan Afr Med J.

